# Fusion sequencing via terminator‐assisted synthesis (FTAS‐seq) identifies *TMPRSS2* fusion partners in prostate cancer

**DOI:** 10.1002/1878-0261.13428

**Published:** 2023-04-13

**Authors:** Ugnė Drazdauskienė, Žana Kapustina, Justina Medžiūnė, Varvara Dubovskaja, Rasa Sabaliauskaitė, Sonata Jarmalaitė, Arvydas Lubys

**Affiliations:** ^1^ Thermo Fisher Scientific Baltics Vilnius Lithuania; ^2^ National Cancer Institute Vilnius Lithuania; ^3^ Institute of Biosciences, Life Sciences Center Vilnius University Lithuania

**Keywords:** fusion transcripts, prostate cancer, RNA sequencing, *TMPRSS2*, transcriptomics

## Abstract

Genetic rearrangements that fuse an androgen‐regulated promoter area with a protein‐coding portion of an originally androgen‐unaffected gene are frequent in prostate cancer, with the fusion between transmembrane serine protease 2 (*TMPRSS2*) and ETS transcription factor ERG (*ERG*) (*TMPRSS2‐ERG* fusion) being the most prevalent. Conventional hybridization‐ or amplification‐based methods can test for the presence of expected gene fusions, but the exploratory analysis of currently unknown fusion partners is often cost‐prohibitive. Here, we developed an innovative next‐generation sequencing (NGS)‐based approach for gene fusion analysis termed fusion sequencing via terminator‐assisted synthesis (FTAS‐seq). FTAS‐seq can be used to enrich the gene of interest while simultaneously profiling the whole spectrum of its 3′‐terminal fusion partners. Using this novel semi‐targeted RNA‐sequencing technique, we were able to identify 11 previously uncharacterized *TMPRSS2* fusion partners and capture a range of *TMPRSS2‐ERG* isoforms. We tested the performance of FTAS‐seq with well‐characterized prostate cancer cell lines and utilized the technique for the analysis of patient RNA samples. FTAS‐seq chemistry combined with appropriate primer panels holds great potential as a tool for biomarker discovery that can support the development of personalized cancer therapies.

## Graphical abstract

The authors developed an innovative NGS‐based approach for gene fusion analysis: fusion sequencing via terminator‐assisted synthesis (FTAS‐seq). FTAS‐seq can be used to enrich the gene of interest while simultaneously profiling its 3′‐terminal fusion partners. Using this novel semi‐targeted RNA‐sequencing technique, the authors identified 11 previously uncharacterized *TMPRSS2* fusion partners and captured a range of *TMPRSS2‐ERG* isoforms.

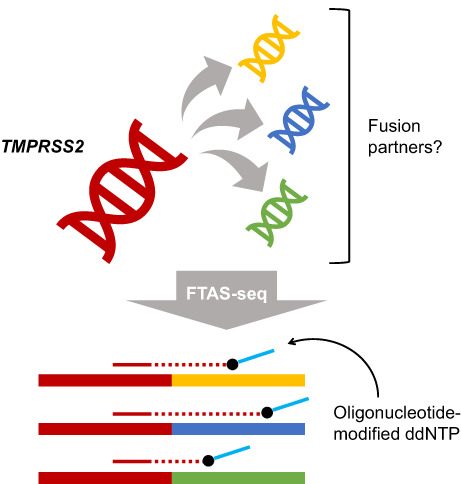

**Abbreviations**
ACN: acetonitrile
BCR: biochemical recurrence
EDTA: ethylenediaminetetraacetic acid
FFPE: formalin‐fixed, paraffin‐embedded
FISH: fluorescence *in situ* hybridization
FTAS‐seq: fusion sequencing via terminator‐assisted synthesis
HPLC: high‐performance liquid chromatography
IHC: immunohistochemistry
NGS: next‐generation sequencing
OTDDN: oligonucleotide‐tethered dideoxynucleotide
PCa: prostate cancer
PSA: prostate‐specific antigen
RIN: RNA integrity number
RT‐PCR: reverse transcription–polymerase chain reaction
RT‐qPCR: reverse transcription–quantitative polymerase chain reaction
TEAAc: triethylammonium acetate
THPTA: tris‐hydroxypropyltriazolylmethylamine
*TMERG*
: *TMPRSS2‐ERG*
UTR: untranslated region

## 1 Introduction

Clinical heterogeneity of prostate cancer (PCa) likely reflects the diversity of the underlying molecular landscape and raises challenges for disease management [1]. Gene fusions are commonly detectable in PCa, with the most frequent type being structural rearrangements between an androgen‐regulated gene and a member of the ETS family transcription factor gene [2, 3]. Typically, fusion events juxtapose androgen‐regulated 5′‐UTR with 3′ protein‐coding parts of oncogenic ETS family genes, resulting in their overexpression. The Cancer Genome Atlas (TCGA) revealed that 53% of patients with PCa had fusions involving ETS family genes [4]. In particular, the *TMPRSS2‐ERG (TMERG)* fusion is the most prevalent somatic fusion event in PCa. The frequency of *TMERG* is variable among different populations: The highest frequency (> 50%) was reported in Caucasians, followed by African American (20–30%) and Asian men (< 20%) [5]. The association of *TMERG* with PCa clinical outcomes remains inconclusive. Some studies have reported the role of *ERG* fusion in PCa cellular growth, tumor progression, and bone metastasis development [6, 7], while others found that patients with fusion‐bearing tumors had not significantly different prognoses than those without [4].

Cancer‐specific fusion transcripts mainly are the result of chromosomal rearrangements, such as translocation, deletion, or isochromosome formation [8]. Chimeric mRNAs can also form because of RNA polymerase read‐through between neighboring genes encoded on the same DNA strand or *trans*‐splicing of pre‐mRNA [9, 10]. Transcription‐induced gene fusions are frequently detected in normal tissues [11], which provides an additional level of molecular complexity in the search for disease‐related biomarkers, nevertheless, in some instances, nonchromosomal fusions, such as *SLC45A3‐ELK4*, may also be involved in pathological processes [12]. Interestingly, different regions of a single tumor may contain distinct patterns of fusion isoforms suggesting that particular gene fusions may emerge independently in different regions of a single organ, as is the case with 17 reported isoforms of *TMERG* in PCa [13, 14]. The prognostic significance of all individual *TMERG* fusion variants is still to be determined.

Fluorescence *in situ* hybridization (FISH), immunohistochemistry (IHC), and reverse transcription–polymerase chain reaction (RT‐PCR) are predominantly used for the detection of fusion genes in clinical settings. Although sensitive, these methods typically test for the presence of already known fusion genes, often leading to false negative results attributed to novel or nontested gene fusions or isoforms. Moreover, low throughput and limited resolution severely limit the diagnostic scope [15]. These weaknesses can be overcome by the application of next‐generation sequencing (NGS), which has already helped to elucidate more than 90% of known fusion genes [16]. However, high‐confidence detection of fusion genes from whole genome or whole transcriptome sequencing data requires extremely deep sequencing, which can be cost‐prohibitive. A minimum of 1000× coverage is necessary to detect low‐abundance fusion transcripts by RNA sequencing [8, 17].

Enrichment for target sequences via hybridization capture or PCR can improve the detection sensitivity of NGS‐based assays. Targeted RNA sequencing employing hybridization probes was shown to increase the diagnostic rate of gene fusion detection from 63% to 76% compared with FISH and RT‐PCR [18]. Among PCR‐based enrichment techniques, anchored PCR chemistry retains the ability to detect previously uncharacterized fusion partners by using a single gene‐specific primer (‘anchor’) and a universal primer embedded in NGS platform‐specific adapters [19]. While both approaches demonstrate high sensitivity and specificity, they involve complex and lengthy manipulations with samples that lead to longer turnaround times as compared to conventional methods.

Here, we describe a new NGS‐based assay to profile the diversity of 3′ fusion partners of target genes by extending a single target‐specific primer and randomly terminating extension products with oligonucleotide‐tethered chain terminators bearing sequencing adapters covalently attached to their nucleobases. This reaction simultaneously produces DNA fragments suitable for short‐read sequencing and labels the resulting molecules with a sequencing adapter. We termed this technique fusion sequencing via terminator‐assisted synthesis or FTAS‐seq. We demonstrated the detection of expected and previously uncharacterized *TMERG* isoforms in the NCI‐H660 cell line and the correct identification of fusion‐negative cases. Moreover, we screened for *TMPRSS2* 3′ fusion partners in prostate tissue samples from PCa patients and discovered 11 new partner genes, as well as a rich diversity of *TMERG* isoforms.

## 2 Materials and methods

### 2.1 Source and culture of prostate cancer cell lines

LNCaP clone FGC (ATCC:CRL‐1740™, RRID:CVCL_1379) and NCI‐H660 (ATCC:CRL‐5813™, RRID:CVCL_1576) cell lines were obtained from ATCC. Both cell lines were authenticated by short tandem repeat (STR) profiling within 3 years before the study and regularly tested for the presence of mycoplasma.

LNCaP cells were cultured in Roswell Park Memorial Institute (RPMI)‐1640 medium supplemented with 10% fetal bovine serum. NCI‐H660 cells were cultured in RPMI‐1640 supplemented with 5% fetal bovine serum, 5 μg·mL^−1^ of insulin, 10 nm of hydrocortisone, 10 μg·mL^−1^ of transferrin, 30 nm of sodium selenite, 10 nm of β‐estradiol, and 2 nm of L‐glutamine. Cells were cultured according to the standard mammalian tissue culture protocols at 37 °C in the presence of 5% CO_2_. All experiments were performed with mycoplasma‐free cells.

### 2.2 RNA extraction from cell lines

Total RNA was purified from prostate cancer cell lines using GeneJET™ RNA Purification Kit (Thermo Fisher Scientific, Waltham, MA, USA) following Mammalian Cultured Cells Total RNA Purification protocol. RNA concentration was measured by NanoDrop™ 2000 Spectrophotometer (Thermo Fisher Scientific). Genomic DNA was removed by treatment with DNase I. Then, RNA was purified using GeneJET™ RNA Cleanup and Concentration Micro Kit (Thermo Fisher Scientific). RNA integrity was assessed by the Agilent 2100 Bioanalyzer system (Agilent Technologies, Santa Clara, CA, USA) using RNA 6000 Nano Kit.

### 2.3 Patient samples

The study used 54 PCa tissue samples (39 *TMERG*‐positive and 15 *TMERG*‐negative as assessed by RT‐qPCR targeting *TMERG* regions spanning *TMPRSS2* exons 1 and 2, and *ERG* exons 1, 2, 3 and 4) collected during 2008–2014 at the Urology Centre of Vilnius University Hospital Santaros Clinic, Lithuania. PCa samples were obtained from PSA‐screened and biopsy‐proven PCa patients treated with radical prostatectomy. The study was approved by the Regional Bioethics Committee (No. 158200‐17‐874‐411), and written informed consent was obtained from all participants. The study methodologies conformed to the standards set by the Declaration of Helsinki.

Biochemical recurrence (BCR) was defined as postoperative PSA levels of 0.2 ng·mL^−1^ and above. Full follow‐up data were available for 52 patients with a mean follow‐up of 3.3 years. Clinico‐pathological and molecular characteristics of the study subsets are provided in Table S1.

Total RNA from tissue was purified using TRIzol™ (Thermo Fisher Scientific) reagent according to the protocol provided by the manufacturer. NanoDrop 2000 Spectrophotometer (Thermo Fisher Scientific) was used to determine RNA concentration and purity.

Total RNA was purified in 2018 from January to March and stored at −80 °C until use. Before manipulations were carried out in this study, RNA quality was assessed by the Agilent 2100 Bioanalyzer system (Agilent Technologies) using RNA 6000 Pico Kit.

### 2.4 Gene expression analysis by RT‐qPCR

*TMPRSS2‐ERG* and *GAPDH* expression levels in cell lines and patient samples were evaluated by reverse transcription–quantitative PCR (RT‐qPCR) analysis. *TMPRSS2‐ERG* fusion transcripts were amplified with the primers specific to *TMPRSS2* exon 1 5′‐CGCGAGCTAAGCAGGAG‐3′ and *ERG* exon 2 5′‐GTCCATAGTCGCTGGAGGAG‐3′. *GAPDH* transcript was amplified with the primer set 5′‐ATTCCATGGCACCGTCAAG‐3′ and 5′‐TTTGGAGGGATCTCGCTCC‐3′. All primers used in this study were synthesized by Metabion International AG. RT‐qPCR reaction mixtures were prepared with 100 ng of total RNA using Power SYBR™ Green RNA‐to‐CT™ 1‐Step Kit (Thermo Fisher Scientific), and each sample was analyzed in duplicate. Cycling was performed following the manufacturer's recommendations. Negative controls without reverse transcriptase were prepared for each sample and multiple no‐template controls were included in each run. Gene expression analysis was performed on QuantStudio™ 7 Flex Real‐Time PCR System using quantstudio™ real‐time pcr software v1.7.1 (Thermo Fisher Scientific).

### 2.5 cDNA synthesis and purification

cDNA was synthesized from LNCaP, NCI‐H660, and clinical samples’ total RNA and used for semi‐targeted RNA‐seq library preparation, as well as for fusion breakpoint validation experiments. Reverse transcription was performed with 500 ng of total RNA using SuperScript™ IV VILO™ Master Mix (Thermo Fisher Scientific) followed by RNA strand hydrolysis by *Escherichia coli* RNase H (Thermo Fisher Scientific). cDNA was then purified using Dynabeads Cleanup Beads (Thermo Fisher Scientific). RT reaction mixtures (20 μL) were mixed with 10 μL of nuclease‐free water, 60 μL of magnetic beads, 60 μL of 96% ethanol, and incubated at room temperature for 10 min. Samples were then placed in the magnetic rack and the supernatant was removed. Beads were washed with 85% ethanol keeping tubes in the magnetic rack. To elute cDNA, beads were resuspended in 10 μL of nuclease‐free water and incubated at 65 °C for 5 min.

### 2.6 Synthesis of oligonucleotide‐tethered dideoxynucleotides (OTDDNs)

All reaction components were added to the reaction mixture as solutions in water unless specified otherwise. A modified oligonucleotide of the sequence 5′‐hexynyl‐AGATCGGAAGAGCACACGTCTG‐biotin‐3′ (ON) was synthesized by Metabion International AG requesting HPLC purification.

5‐(3‐(2‐azidoacetamido)prop‐1‐ynyl)‐2′,3′‐dideoxycytidine‐5′‐triphosphate or 5‐(3‐(2‐azidoacetamido)prop‐1‐ynyl)‐2′,3′‐dideoxyuridine‐5′‐triphosphate (3 eq.) solution was added to 5′‐hexynyl modified oligonucleotide (200–210 nmol) solution in sodium phosphate buffer (1 mL, 100 mm, pH 7). A premixed solution of CuSO_4_ (100 mm, 12 eq.) and THPTA (250 mm, 5 eq. to CuSO_4_) was then added to the reaction mixture, followed by the addition of sodium ascorbate (1 m, 50 eq. to CuSO_4_). The reaction mixture was stirred for 20 min at 42 °C and quenched with 0.5 m EDTA‐Na_2_ solution (1 mL, pH 8). The products were purified by C18 reversed‐phase chromatography using 100 mm TEAAc/ACN (11–18%) as eluent and desalted using water/ACN (0–100%) as eluent.

The oligonucleotide‐modified ddC^ON^TP was obtained with 39% (82 nmol) yield. HRMS (ESI^−^): calculated monoisotopic mass for [M]: 7916.345; found: 7916.342. The oligonucleotide‐modified ddU^ON^TP product was obtained with 34% (67 nmol) yield. HRMS (ESI^−^): calculated monoisotopic mass for [M]: 7917.329; found: 7917.326. The synthesis scheme of oligonucleotide‐tethered dideoxynucleotides is shown in Fig. S1.

### 2.7 Semi‐targeted RNA‐seq library preparation

To prepare semi‐targeted RNA‐seq libraries from cDNA inputs, Thermo Sequenase (Thermo Fisher Scientific) enzyme, which is capable to extend primer and incorporate OTDDNs, was employed. Each sample was analyzed in duplicate. Primer extension reaction mixtures contained 8 μL of purified cDNA (corresponding to ~ 500 ng of initial total RNA), 1× Thermo Sequenase Reaction Buffer, 0.05 μm of *TMPRSS2* exon 1 specific primer 5′‐TAGGCGCGAGCTAAGCAGGAG‐3′, 1 μm of dATP and dGTP each, 0.9 μm of dTTP and dCTP each, 0.1 μm of ddC^ON^TP and ddU^ON^TP each, 40 U of Thermo Sequenase and nuclease‐free water up to 20 μL final volume. Second strand synthesis conditions were as follows: denaturation at 95 °C for 4 min, followed by 15 cycles of denaturation at 95 °C for 1 min, annealing at 63 °C for 30 s, extension at 72 °C for 1 min, and final extension at 72 °C for 5 min. Primer extension products were then purified following conditions described for cDNA purification, except that the elution volume was increased to 22 μL of nuclease‐free water.

To amplify NGS libraries and to increase target detection specificity, indexing nested‐PCR was performed. Fragments were amplified with an indexing primer set in which one primer was specific to the *TMPRSS2* target sequence: i5 primer 5′‐AATGATACGGCGACCACCGAGATCTACACTCTTTCCCTACACGACGCTCTTCCGATCTGGAGGCGGAGGGCGAGGG‐3′, and i7 primer 5′‐CAAGCAGAAGACGGCATACGAGAT[8nt_index]GTGACTGGAGTTCAGACGTGTGCTCTTCCGATCT‐3′. Indexing nested‐PCR reaction mixture contained 20 μL of purified primer extension products, 1× Invitrogen™ Collibri™ Library Amplification Master Mix (Thermo Fisher Scientific), 1 μm of indexing primers each, 20 U of 3′‐5′ exonuclease‐deficient Phusion polymerase, and nuclease‐free water up to 50 μL final volume. Amplification conditions were as follows: denaturation at 98 °C for 30 s, followed by 20 cycles of denaturation at 98 °C for 10 s, annealing at 60 °C for 30 s, extension at 72 °C for 1 min, and final extension at 72 °C for 1 min. PCR products were then purified using Dynabeads Cleanup Beads following a double binding protocol. 50 μL of samples was mixed with 40 μL of magnetic bead suspension and incubated at room temperature for 5 min. Samples were then placed in the magnetic rack, the supernatant was removed, and beads were resuspended in 50 μL of nuclease‐free water. For the second binding, samples were mixed with 45 μL of fresh beads suspension and incubated at room temperature for 5 min. After incubation samples were placed in the magnetic rack, the supernatant was removed, and beads were washed with 85% ethanol. Beads were then resuspended in 22 μL of nuclease‐free water and incubated at room temperature for 1 min to elute final libraries. Fragment size distribution and concentration were evaluated by the Agilent Fragment Analyzer system (Agilent Technologies) using HS NGS Fragment Kit.

To generate enough material for sequencing, samples were reamplified with Invitrogen Collibri Library Amplification Master Mix with Primer Mix (Thermo Fisher Scientific) in a 50 μL reaction for 3–7 cycles according to the manufacturer's protocol. Final libraries were purified using Dynabeads Cleanup Beads following double binding protocol and analyzed by the Agilent Fragment Analyzer system (Agilent Technologies) with HS NGS Fragment Kit. Finally, libraries were quantified with the Invitrogen Collibri Library Quantification Kit (Thermo Fisher Scientific).

### 2.8 Next‐generation sequencing

NGS libraries were mixed with 20% of PhiX Control v3 (Illumina, San Diego, CA, USA). Semi‐targeted RNA‐seq libraries were sequenced on the Illumina™ MiSeq™ instrument using MiSeq Reagent Kit v2 (300‐cycle) at 2 × 150 bp PE mode or MiSeq Reagent Kit v3 (600‐cycle) at 2 × 300 bp PE mode. Sequencing depth varied from 0.2 to 0.6 m reads per sample.

### 2.9 Sequencing data analysis and detection of fusion transcripts

snakemake workflow manager v6.1.0 [20] was used to implement the RNA‐seq data analysis pipeline. Raw PE reads were processed with bbduk tool from bbmap suit v37.90 [21] to trim adaptor sequences and exclude low‐quality reads and poor‐quality bases using the following parameters: minlength 50, qtrim r, trimq 15, tpe tbo, maxns 1, hdist 1, ktrim r, k 23, mink 11. Processed reads were mapped to a reference human genome version hg19 using the Spliced Transcripts Alignment to a Reference (star v2.5.3) aligner [22] with additional settings to capture discordant read pairs and chimeric read alignments “‐‐chimOutType SeparateSAMold SoftClip ‐‐chimSegmentMin 10 ‐‐chimJunctionOverhangMin 10 ‐‐chimScoreMin 1 ‐‐chimScoreDropMax 30 ‐‐chimScoreJunctionNonGTAG 0 ‐‐chimScoreSeparation 1 ‐‐alignSJstitchMismatchNmax 5 ‐1 5 5 ‐‐chimSegmentReadGapMax 3”. Mapping quality was assessed with picard v2.22.3 [23], rseqc v2.6.2 [24], and qualimap v2.2.1 [25]. For transcript counts and biotype calculations, qorts v1.3.0 [26] was used. Aligned BAM files were deduplicated using Picard MarkDuplicates with REMOVE_DUPLICATES = True and optical_dublicate_pixel_distance = 100.

Fusion transcripts were detected with a command‐line tool arriba v1.2.0 [27] using deduplicated BAM files and chimeric reads from STAR aligner with the following command: ‘arriba ‐x deduplicated.bam ‐c Chimeric.out.sam ‐o arriba_fusions.tsv ‐O arriba_fusions.discarded.tsv ‐b blacklist_hg19_hs37d5_GRCh37_2018‐11‐04.tsv.gz ‐a hg19.fa ‐g hg19.gtf ‐T ‐P’. Fusion visualization was performed with Arriba Rscript: ‘arriba_draw_fusions.r ‐‐fusions=arriba_fusions.tsv ‐‐alignments=deduplicated.bam ‐‐output=arriba_fusions.pdf ‐‐annotation=hg19.gtf ‐‐cytobands=cytobands_hg19_hs37d5_GRCh37_2018‐02‐23.tsv ‐‐proteinDomains=protein_domains_hg19_hs37d5_GRCh37_2019‐07‐05.gff3’. Files with blacklist regions, cytobands, and protein domains are provided with the download package of Arriba [28].

The schematic depiction of the data analysis workflow is given in Fig. S2.

### 2.10 Fusion breakpoint validation by Sanger sequencing

To validate breakpoint sequences patient samples were reverse transcribed and fused fragments were amplified using *TMPRSS2* exon 1 specific primer 5′‐TAGGCGCGAGCTAAGCAG‐3′ and reverse primer specific to 3′ fusion partner (Table S2). Amplification mixtures contained 8 μL of purified cDNA (corresponding to ~ 500 ng of initial total RNA), 1× Phusion Hot Start II High‐Fidelity PCR Master Mix (Thermo Fisher Scientific), 0.5 μm of forward and reverse primers each, and nuclease‐free water up to 20 μL final volume. PCR conditions: denaturation at 98 °C for 30 s, followed by 35 cycles of denaturation at 98 °C for 10 s, annealing at 59–62 °C (depends on primer Tm °C) for 30 s, extension at 72 °C for 15 s, and final extension at 72 °C for 5 min. Amplified fusion isoforms were separated by agarose gel electrophoresis on high resolution 2% MetaPhor™ Agarose (Lonza, Basel, Switzerland), and DNA was extracted using GeneJET Gel Extraction and DNA Cleanup Micro Kit (Thermo Fisher Scientific). Purified PCR fragments were cloned using CloneJET™ PCR Cloning Kit (Thermo Fisher Scientific), and ligation mixtures were then used for *E. coli* DH10B transformation. Plasmids were purified with GeneJET Plasmid Miniprep Kit (Thermo Fisher Scientific) and sequenced with BigDye™ Terminator v3.1 Cycle Sequencing Kit on 3130xL Genetic Analyzer system (Thermo Fisher Scientific). Obtained sequences were analyzed using Vector NTI Advance Software v11.5.4 (Thermo Fisher Scientific).

## 3 Results

### 3.1 FTAS‐seq captures unknown RNA 3′‐terminal sequences nearby a defined target site

**Fig. 1:**
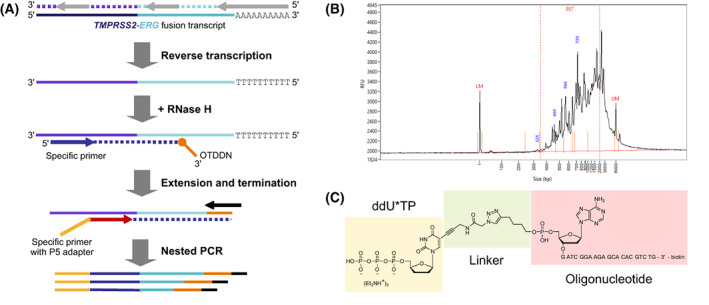
Overview of FTAS‐seq technique. (A) Library preparation workflow for the detection of *TMERG* fusion transcripts in the human transcriptome. (B) Typical FTAS‐seq library trace. (C) The structure of oligonucleotide‐tethered dideoxynucleotides as exemplified by oligonucleotide‐modified ddUTP. The structure was drawn using the chemdraw software (PerkinElmer Informatics, Waltham, MA, USA).

To analyze *TMPRSS2* 3′ fusion partner sequences, we developed FTAS‐seq (Fig. 1A), which utilizes a nucleotide‐mediated adapter addition technology for the preparation of sequencing‐ready molecules by the extension of a single site‐specific primer. Firstly, total RNA was reverse transcribed using random and oligo(dT) primers, then RNase H was used to hydrolyze the RNA strand in an RNA‐cDNA hybrid. We designed a primer specific to *TMPRSS2* (RefSeq NM_005656) exon 1 to target the 5′ end of the transcript. The semi‐targeted approach requires knowing only a small fragment of the target gene to design a primer and enables the detection of adjacent 3′‐terminal regions, in our case *ERG* sequences (RefSeq NM_004449.4), without *a priori* knowledge. Second cDNA strand synthesis was initiated from a target‐specific primer and utilized DNA polymerase, which is able to incorporate dideoxynucleotides. Base‐modified dideoxynucleotides contained a synthetic oligonucleotide, which served as a universal priming site to amplify labeled cDNA fragments. Next, *TMPRSS2* exon 1‐specific primer, which hybridizes closer to the fusion breakpoint and contains a full‐length Illumina P5 adapter, was used in the library amplification step to increase *TMERG* detection specificity and to add Illumina P5 adapters to amplified fragments. A full‐length P7 adapter was introduced through indexing primers complementary to OTDDN oligonucleotide. After amplification short fragments were removed by applying size‐selection purification (Fig. 1B), and libraries were subjected to standard Illumina paired‐end sequencing. The forward sequencing read (R1) contained fusion transcripts' 5′ end sequences starting from *TMPRSS2* exon 1 while the reverse read (R2) contained *ERG* sequences starting from random positions.

The use of click chemistry as a means of adapter addition was previously reported [29]. Here, we executed click reactions to generate oligonucleotide‐modified dideoxynucleotides prior to their incorporation into the nascent DNA strand (Fig. 1c). We obtained conjugates of correct mass and 98% purity with > 30% yield. An essential requirement for these compounds is the compatibility of the unnatural linker with DNA polymerases to enable the use of the attached oligonucleotide as a priming site. We optimized the structure of the linker and identified polymerases able to use OTDDNs as substrates, as well as polymerases able to perform read‐through [30]. These findings pave the way for straightforward DNA labelling by any desired oligonucleotide irrespective of the sequence context of the template.

### 3.2 FTAS‐seq detects the expected *TMERG* isoforms in prostate cancer cell line

**Fig. 2:**
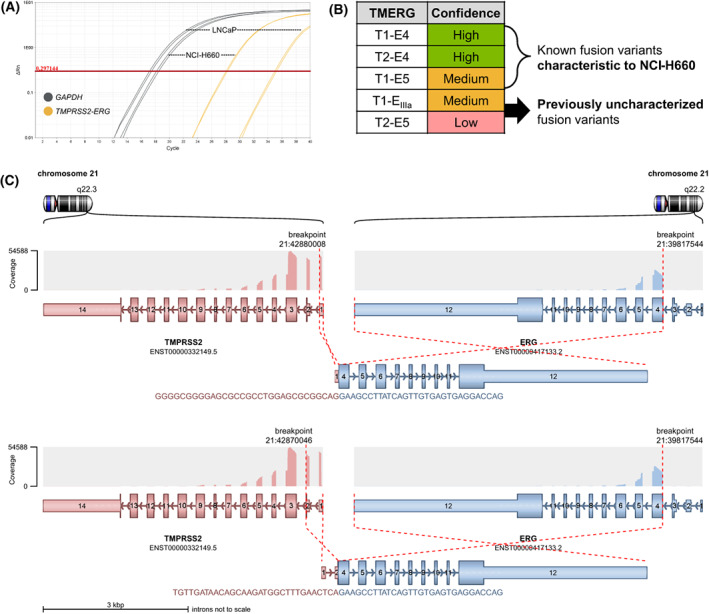
Detection of *TMERG* fusion transcripts in prostate cancer cell lines. (A) *TMERG* and *GAPDH* expression in NCI‐H660 (*n* = 2) and LNCaP (*n* = 2) total RNA assessed by RT‐qPCR. (B) *TMERG* isoforms detected by FTAS‐seq in NCI‐H660 RNA with high (green cells), medium (yellow cells), and low (red cells) confidence scores. Confidence scores in Arriba are influenced by the number of supporting reads and other parameters, such as the level of background noise in a given gene [27]. E_IIIa_ is an *ERG* intronic sequence after exon 3. (C) The structure of T1‐E4 and T2‐E4 fusion transcripts detected in NCI‐H660 RNA. The visualization was created using the Arriba Rscript arriba_draw_fusions.R.

To assess whether the semi‐targeted RNA‐seq approach is sufficiently specific and sensitive to detect rare fusion events in human transcriptome, we prepared FTAS‐seq libraries from total RNA purified from prostate cancer cell lines. We selected the NCI‐H660 cell line as a *TMERG*‐positive [13, 31] and LNCaP as a negative control [31]. Firstly, we evaluated *TMERG* expression levels by RT‐qPCR and observed that *TMERG* was ~ 1000‐fold less abundant than housekeeping *GAPDH* transcript in NCI‐H660 cells (Fig. 2A). Although we obtained *TMERG* signal for LNCaP cells as well, melt curve analysis indicated that these were likely nonspecific amplification products. Afterwards, we sequenced FTAS‐seq libraries prepared from 0.5 μg of the same total RNA. On average, 73.14 ± 0.34% of obtained reads mapped to hg19 in NCI‐H660 samples. To detect gene fusions from semi‐targeted RNA‐seq data, we employed a command‐line tool Arriba, which evaluates the reliability of each fusion according to an internal confidence scoring algorithm [27]. We considered fusion isoforms as putative true variants if they met the Arriba criteria and were detected in both technical replicates. Five *TMERG* fusions were found in NCI‐H660 samples (Fig. 2B,C) of which three were previously reported [13]. In this study, we named fusion transcripts by exon numeration according to *TMPRSS2* and *ERG* transcripts structure. In NCI‐H660 libraries we also identified T1‐E_IIIa_ and T2‐E5 fusion transcripts, which were not validated in NCI‐H660 transcriptome before, although were reported in PCa patient samples [13]. All identified *TMERG* variants, excluding T1‐E_IIIa_, which is identical to T1/E_IIIc_4 described by Clark *et al*. [13], form between corresponding *TMPRSS2* and *ERG* exons while T1‐E_IIIa_ contains partial *ERG* intron 3 sequence followed by exon 4.

To assess whether FTAS‐seq data are affected by technical noise and would correctly detect *TMERG*‐negative cases, we prepared FTAS‐seq libraries from total RNA extracted from LNCaP cells. 81.32 ± 0.68% of obtained reads mapped to hg19. The majority of reads in LNCaP samples mapped to wild‐type *TMPRSS2* gene and no chimeric transcripts were detected. Experiments with PCa cell lines indicate that FTAS‐seq is a specific method for the detection of rare *TMERG* transcripts, which enables distinguishing fusion‐positive and ‐negative cases.

### 3.3 The analysis and validation of *TMPRSS2* gene fusions in PCa tumor samples

Following successful proof‐of‐principle experiments in cell lines, we applied the semi‐targeted RNA‐seq approach to analyze the diversity of *TMPRSS2* fusion partners in PCa samples. The study cohort contained *TMERG*‐positive and *TMERG*‐negative cases as assessed by conventional PCR‐based methods (Table S1). After sequencing, obtained reads were mapped to the hg19 reference genome—alignment rate varied from 22.90% to 89.04%, and this correlated with the quality of RNA used for library preparation (RIN 2.2–8.2). The analysis of NGS data also showed that target detection specificity and the number of contaminating non‐*TMPRSS2* reads are related to the integrity of RNA input. Importantly, quality differences did not force us to exclude samples from the analysis—meaningful information was retrieved in all cases. Our results show that the semi‐targeted RNA‐seq approach may be applied to analyze even highly degraded RNA. Nevertheless, a separate validation is needed to verify the compatibility with FFPE samples as FFPE‐derived RNA tends to be not only fragmented but also chemically modified.

Across a total cohort, FTAS‐seq detected chimeric transcripts in 43 samples (77%) of which 38 (70%) contained *TMERG* fusion transcripts (Table S3). In comparison, 39 samples (72%) were previously reported as *TMERG*‐positive by RT‐qPCR and contained at least one of the variants T1‐E4 and/or T2‐E4 or T1‐E2. To assess the overall concordance of FTAS‐seq and RT‐qPCR we compared the number of fusion isoforms detected by both methods. FTAS‐seq correctly detected *TMERG* transcripts in 32 (82%) samples and nine isoforms were missed in 7 (18%) samples. Contradictory samples were then processed to Sanger sequencing to validate *TMERG* status. The results showed that three isoforms were indeed missed by FTAS‐seq likely due to their low expression levels and/or interference from the wild‐type *TMPRSS2* transcript originating from the nontumor cells. Six other isoforms were not detected neither by FTAS‐seq nor by Sanger sequencing indicating the false positives of RT‐qPCR.

### 3.4 Novel 3′ fusion partners of *TMPRSS2*

**Fig. 3:**
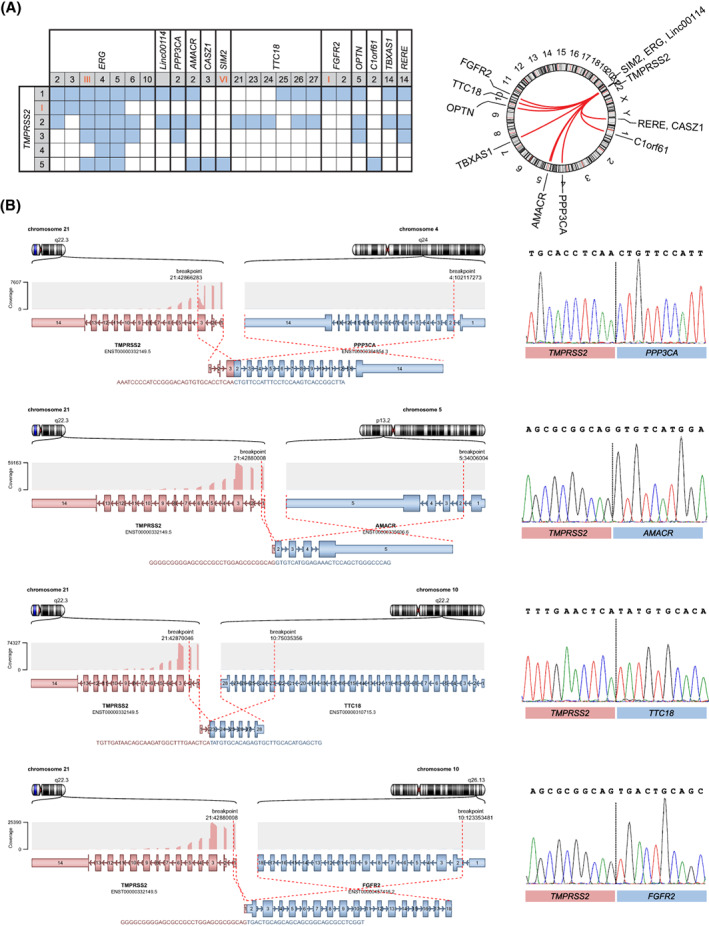
*TMPRSS2* fusion transcripts detected in clinical prostate tissue RNA samples. (A) Left: Unique fusion transcripts detected in patient samples by FTAS‐seq and Sanger sequencing. Numbers in the table indicate the exons and orange Roman numerals stand for the respective introns at the breakpoint sites. Blue cells indicate the detected fusion transcripts. Right: A Circos plot illustrates the chromosomal locations of *TMPRSS2* fusion partners identified in this study. (B) Examples of interchromosomal fusions (*TMPRSS2*‐*PPP3CA*, *TMPRSS2‐AMACR*, *TMPRSS2‐TTC18*, and *TMPRSS‐FGFR2*) that were discovered in this study. Each chimeric transcript was visualized with Arriba Rscript arriba_draw_fusions.R. Breakpoints of novel fusions were validated by Sanger sequencing.

The semi‐targeted RNA‐seq approach enabled us to analyze the variety of 3′ fusion partners associated with the *TMPRSS2* gene. Across 54 PCa samples, we detected 11 novel *TMPRSS2* fusion partners (*Linc00114*, *PPP3CA*, *AMACR*, *CASZ1*, *SIM2*, *TTC18*, *FGFR2*, *OPTN*, *C1orf61*, *TBXAS1*, *RERE*) bearing 21 different breakpoint sequence (Fig.3, Table S4). All novel variants were detected in individual samples, except for *AMACR* fusions that were found at a higher frequency. Eight (15%) samples contained *TMPRSS2‐AMACR* breakpoints, which form between *TMPRSS2* exon 1 or 5 and *AMACR* exon 2. The variety of chimeric transcripts showed that fusions may contain junctions of different structures. For example, 18 out of 21 new variants contained exon‐exon junctions, one variant (T5‐S_VI_) consisted of *TMPRSS2* exon fused with the inverted sequence of *SIM2* intron 6, and two variants consisted of *TMPRSS2* exons fused with noncoding RNA *Linc00114* sequences. FTAS‐seq analysis indicates that PCa patient samples exhibit a wide diversity of *TMERG* and other chimeric transcripts, which cannot be detected by RT‐qPCR without prior knowledge about both partner genes.

To verify novel transcripts, we processed samples to Sanger sequencing. *PPP3CA*, *AMACR*, *TTC18*, *Linc00114*, *FGFR2*, *TBXAS1*, and *RERE* were identified as valid *TMPRSS2* 3′‐terminal fusion partners (Fig. 3). We were not able to analyze *CASZ1*, *SIM2*, and *C1orf61* fusion isoforms due to insufficient amount of input RNA. During validation, we found a large part of gene fusions detected by FTAS‐seq, as well as additional previously uncaptured isoforms. Sanger sequencing revealed 15 fusion variants that were not found by FTAS‐seq and could not have been detected by RT‐qPCR targeting T1‐E4. Our results show that the majority of novel fusion transcripts identified by semi‐targeted RNA‐seq, although rare, are true variants characteristic to PCa (Table S3).

### 3.5 Bioinformatic prediction of actionable gene fusions

Employing semi‐targeted RNA‐seq we identified a great structural variety of fusion transcripts in PCa patient samples. By using bioinformatic tools, we attempted to predict the functionality of the resulting chimeric proteins. Some fusion events, such as T3‐P2, T5‐A2, T2‐TTC23, and T2‐TTC24, do not alter reading frames. These chimeras may encode functional protein domains and may retain the biological functions of a 3′ fusion partner. Androgen‐regulated *TMPRSS2* promoter leads to overexpression of these proteins in prostate tissue. Another part of fusion transcripts has an unclear reading frame, for example, T1‐A2 and T1‐F2, causing an uncertain effect on protein level. Such ambiguities might be caused by the inability to predict the expressed isoform of a 3′ fusion partner that can have an alternative reading frame. Many isoforms were marked as unclear in our dataset indicating that complementary techniques, such as long‐read sequencing, are needed to resolve such ambiguities. The third category of fusions leads to the loss of reading frame. If such rearrangements occur at the genomic level, this might indicate the loss of function of corresponding genes. Alternatively, these fusions may function as noncoding RNA. Bioinformatic predictions can assist in understanding the clinical importance and pathology of fusion transcripts in PCa, although more comprehensive experimental validation is needed to verify the anticipated effects.

## 4 Discussion

In the era of precision oncology, the detection of fusion genes that often play driver roles in tumorigenesis [32] is critical to improve diagnosis and personalize treatment. Moreover, recent reports indicate that even similar clinical phenotypes might substantially differ at the molecular level, implying different drug targets, thus the understanding of molecular features is crucial for precise cancer therapy [33]. Here, we have demonstrated the concept of semi‐targeted RNA sequencing and identified a plethora of *TMPRSS2* 3′‐terminal fusion partners in PCa. We have previously shown that the introduction of sequencing adapters via enzymatic incorporation of base‐modified dideoxynucleotides improves NGS library preparation [30, 34]. This work further expands the applicability of OTDDNs and suggests a semi‐targeted sequencing technique for transcriptomic analyses.

Although long‐read sequencing technologies can detect structural variations in DNA and splice isoforms in RNA [35], reliable and cost‐effective approaches employing short‐read sequencers are still of interest given their accuracy, wide adoption, and support by a broad range of data analysis tools. FTAS‐seq developed in this work offers similar advantages to anchored PCR [19], including agnostic detection of fusion partners by targeting only one known gene. In addition, FTAS‐seq provides a substantially simpler workflow and compatibility with highly degraded RNA that is frequently obtained from FFPE‐derived biosamples.

The fusion of *TMPRSS2* with *ERG* is by far the single most common fusion gene found in solid tumors [2, 36]. Due to its prevalence and the availability of fusion‐positive cell line models, the relevance of *TMERG* for the pathogenesis of PCa has been widely studied. Remarkably, these analyses revealed either positive, negative, or no association between the presence of *TMERG* mRNA and the clinical significance, progression, or aggressiveness of PCa [37]. Limitations of conventional gene fusion detection techniques may be at least in part responsible for these discrepancies and the ability to profile the whole spectrum of *TMPRSS2* fusion partners may greatly facilitate the elucidation of their clinical significance.

High‐throughput sequencing increased the number of known gene fusions to more than 30 000 [38], with approximately 10 000 fusion transcripts identified in normal tissues [11], which raises the question of whether newly detected fusions are clinically important or are random events [39]. The analysis of 2727 candidate fusion genes reported in PCa revealed that most (76%) of genes fuse to a single partner while genes that are likely oncogenic drivers fuse to multiple partners, e.g., the *TMPRSS2* gene was associated with 23 partners. Interestingly, most fusion partners of *TMPRSS2* (65%) were found to be located on different chromosomes [40]. Likewise, our study identified 11 new *TMPRSS2* fusion partners, nine (82%) of which are from different chromosomes. Most of the identified fusions were detected in individual samples while *TMPRSS2‐AMACR* transcripts were detected in eight (15%) cases. Alpha‐methylacyl‐CoA racemase (AMACR) is an enzyme involved in bile acid biosynthesis and peroxisomal beta‐oxidation of branched‐chain fatty acids [41]. Previous studies reported the overexpression of *AMACR* at both protein and mRNA levels in cancerous prostatic tissues [42]. Notably, the chimeric *TMPRSS2‐AMACR* transcript might be a result of trans‐splicing between *TMPRSS2* and *AMACR* pre‐mRNAs as both transcripts are abundant in PCa. To determine the origin of this transcript, additional analysis on a DNA level is needed.

Fusion transcripts between two genes in‐frame are translated into fusion proteins that may act as potent oncogenic drivers [43]. In this study, we identified in‐frame fusions between *TMPRSS2* and *AMACR*, *PPP3CA*, and *TTC18*. *PPP3CA* encodes the catalytic subunit A of a calcium‐dependent protein phosphatase calcineurin. Previous studies indicated a pro‐tumorigenic role of calcineurin signaling in prostate and other types of cancers [44]. *TTC18* encodes cilia‐ and flagella‐associated protein 70, which is a regulator protein of the outer dynein arms strongly expressed in the human testis. Loss of *TTC18* function was found to be responsible for multiple morphological abnormalities of the sperm flagella [45]. In our study in‐frame isoforms of *TMPRSS2‐TTC18* retain only a small fraction of *TTC18* exons, which likely do not exhibit their biological function, thus rearrangement probably leads to the knockout of *TTC18*. Collectively, our findings open doors for the consideration and further exploration of additional molecular mechanisms of PCa progression.

We were not able to validate *TMPRSS2* fusions with *CASZ1*, *SIM2*, and *C1orf61* due to the insufficient amount of RNA; however, all these genes are known to play roles in pathologic conditions, including cancers. *CASZ1* encodes a zinc finger transcription factor whose dysregulated expression was linked to the pathobiology of neuroblastoma [46], glioma [47], and hepatocellular carcinoma, and aberrant fusion transcripts of *CASZ1* were reported in colorectal and bladder cancers [48]. To date, no reports are linking *CASZ1* to PCa. *SIM2* encodes proteins belonging to a family of transcriptional repressors, which are known to be involved in the pathogenesis of solid tumors, including PCa. *SIM2* was previously found to be upregulated in PCa and proposed as a biomarker of aggressive disease [49, 50]. *C1orf61*, or *CROC‐4*, encodes a transcriptional activator of c‐fos proto‐oncogene promoter [51]. C1orf61 was reported to act as a tumor activator and promote metastasis in human hepatocellular carcinoma [52]. This study for the first time indicates the potential role of *CASZ1* and *C1orf61* in PCa and reports the fusions of these genes with *TMPRSS2*.

There are a few alternative NGS‐based techniques for fusion detection available in the market based either on PCR, semi‐targeted PCR, or hybridization capture. The published benchmarking results indicate that all three approaches generate high‐quality results. PCR‐based methods show the lowest limit of detection, while semi‐targeted PCR and hybridization capture techniques are superior for the discovery of uncommon or novel fusion partners [53]. Notably, long‐read sequencing, currently offered by Oxford Nanopore Technologies [54] and Pacific Biosciences [55], can resolve multi‐exon isoforms and accurately detect fusions as reads span the full length of transcripts, however currently these technologies are still more expensive than short‐read sequencing. Moreover, there are few computational tools for the detection of structural variation in long‐read data and these tools typically can characterize only a subset of structural variants [56]. In principle, FTAS‐seq can be adapted for long‐read library preparation by changing the adapter sequences and adjusting the fragment length by reducing the OTDDN to dNTP ratio at the second cDNA strand synthesis step (see Section 2). This might be useful to capture breakpoints that occur at a longer distance from the target site than can be reliably captured by our technique using short reads. The main technical characteristics of FTAS‐seq and other technologies for fusion detection are summarized in Table S5. Notably, FTAS‐seq provides the fastest sample preparation workflow given that OTDDN incorporation eliminates the need for cDNA fragmentation and adapter ligation steps. Technologically, FusionPlex and QIAseq RNAscan are the most similar products to the FTAS‐seq developed in this study. In terms of performance, the FusionPlex assay is known to be susceptible to low‐quality inputs while QIAseq RNAscan panels were shown to generate many false positive calls [57]. This indicates that although semi‐targeted amplification is a very attractive approach for fusion analysis, currently available products are suboptimal.

## 5 Conclusion

The profiling of RNA fusions expands the therapeutic landscape of cancerous tumors. The technique developed in this work allows simple and cost‐effective high‐throughput profiling of 3′‐terminal fusion partners of any cancer‐related genes of interest. The developed technique was 100% specific and showed a sensitivity of 95% as validated by RT‐qPCR. Further development of FTAS‐seq might include the exploration of multiplexing options (with the possibility to design a panel) and tailoring the protocol for lower RNA input amounts. Profiling of 5′‐terminal fusion partners is also possible through the intermediate double‐stranded cDNA synthesis step. In general, this novel method holds great potential to improve clinically relevant gene fusion detection for higher applicability of modern cancer therapies targeting *ALK*, *ROS1*, *RET*, and other gene fusions [58]. Besides, clinically relevant isoforms might contribute to the molecular biomarker toolbox that can be further explored for noninvasive detection opportunities [59].

## Author contributions

UD performed the experiments, analyzed the data, and wrote the manuscript. ŽK designed and supervised the research, and co‐wrote the manuscript. JM synthesized oligonucleotide‐tethered dideoxynucleotides used in this study. VD analyzed NGS data. RS and SJ contributed with patient material and clinical data. RS performed the RT‐qPCR evaluation of *TMPRSS2‐ERG* status in patient samples. SJ and AL conceived the original idea for this research. All authors revised the manuscript and provided comments.

## Conflict of interest

The authors declare the following competing interests: UD, ŽK, JM, VD, and AL are employees of Thermo Fisher Scientific Baltics. A patent covering oligonucleotide‐tethered nucleotides and their uses is pending.

## Supporting information


**Fig. S1.** The scheme of oligonucleotide‐tethered dideoxynucleotide (OTDDN) synthesis.
**Fig. S2.** The flowchart of NGS data analysis to detect fusion transcripts.
**Table S1.** Clinical–pathological characteristics of the study cohort.
**Table S2.** Reverse primers used in end‐point PCR to validate novel breakpoints.
**Table S4.** Fusion breakpoint sequences detected by FTAS‐seq.
**Table S5.** The comparison of the main technical characteristics of the RNA fusion detection methods.

**Table S3.** The variety of fusion transcripts detected in individual patient samples by FTAS‐seq, RT‐qPCR and Sanger sequencing.

## Data Availability

The data that support the findings of this study are openly available in the NCBI BioProject database at https://www.ncbi.nlm.nih.gov/bioproject/, reference number PRJNA776133.
