# Supplementary material for: Fusion sequencing via terminator‐assisted synthesis (FTAS‐seq) identifies TMPRSS2 fusion partners in prostate cancer
Source: Mol Oncol. 2023 Apr 13;17(6):993–1006. doi: 10.1002/1878-0261.13428 (PMC10257418; doi:10.1002/1878-0261.13428)
Supplement: Supplementary file 1 — Fig. S1. The scheme of oligonucleotide‐tethered dideoxynucleotide (OTDDN) synthesis. Fig. S2. The flowchart of NGS data analysis to detect fusion transcripts. Table S1. Clinical–pathological characteristics of the study cohort. Table S2. Reverse primers used in end‐point PCR to validate novel breakpoints. Table S4. Fusion breakpoint sequences detected by FTAS‐seq. Table S5. The comparison of the main technical characteristics of the RNA fusion detection methods. [file MOL2-17-993-s002.docx]

**Supplementary Information**

Fusion sequencing via terminator-assisted synthesis (FTAS-seq) identifies *TMPRSS2* fusion partners in prostate cancer

Ugnė Drazdauskienė^1,4^, Žana Kapustina^1,4,*^, Justina Medžiūnė^1^, Varvara Dubovskaja^1^, Rasa Sabaliauskaitė^2^, Sonata Jarmalaitė^2,3^, Arvydas Lubys^1,*^

^1^ Thermo Fisher Scientific Baltics, V. A. Graičiūno str. 8, Vilnius 02241, Lithuania

^2^ National Cancer Institute, Santariškių str. 1, Vilnius 08406, Lithuania

^3^ Institute of Biosciences, Life Sciences Center, Vilnius University, Saulėtekio av. 7, Vilnius 10257, Lithuania

^4^ These authors contributed equally to this work.

* Correspondence to Ž.K. or A.L. E-mail: zana@olimpiados.lt; arvydas.lubys@thermofisher.com

# 1. Supplementary Figures

**
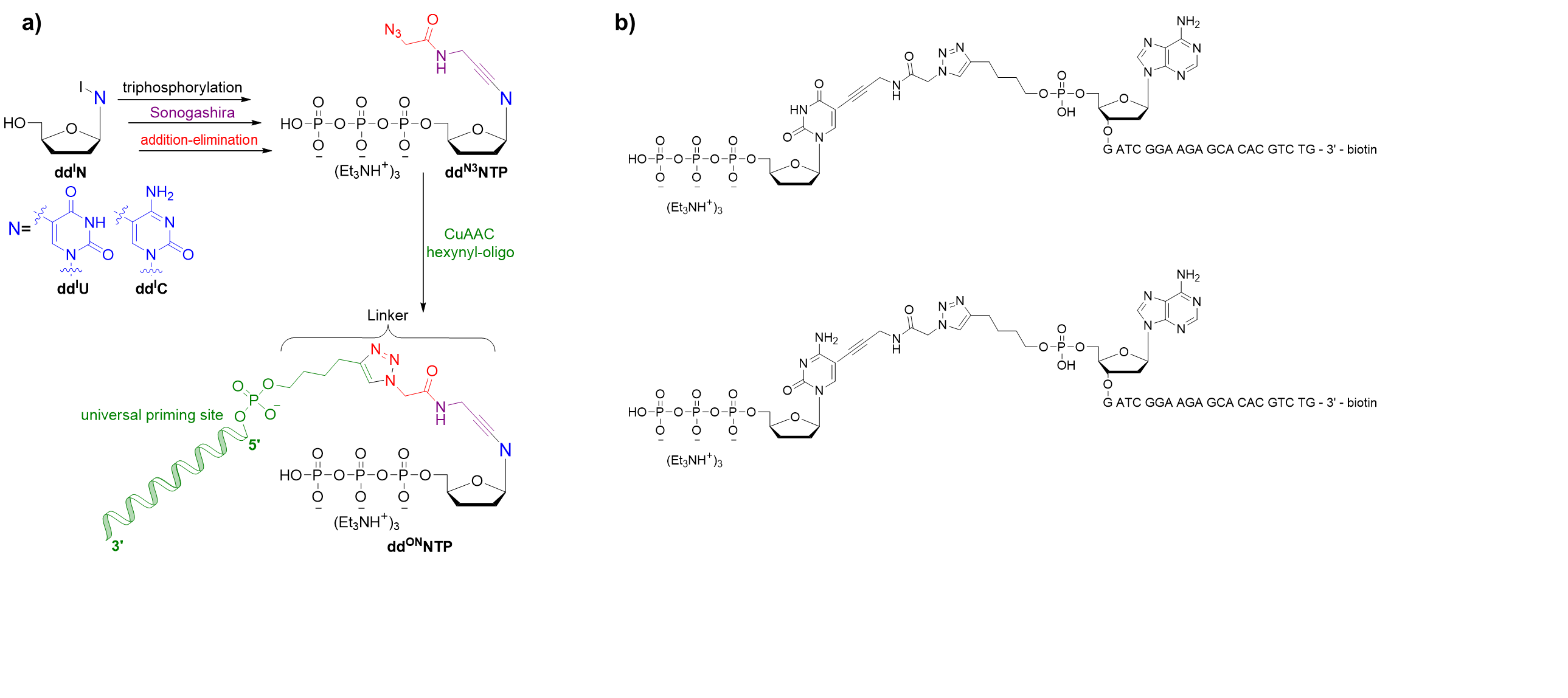
**

**Figure S1.** **The scheme of oligonucleotide-tethered dideoxynucleotide (OTDDN) synthesis. (A)** The hexynyl-modified oligonucleotide is conjugated to azide-modified dideoxynucleotides using click chemistry. (**B)** The structures of OTDDNs used in this work for the semi-targeted RNA-seq library preparation.

Chemical structures were drawn using the ChemDraw software.


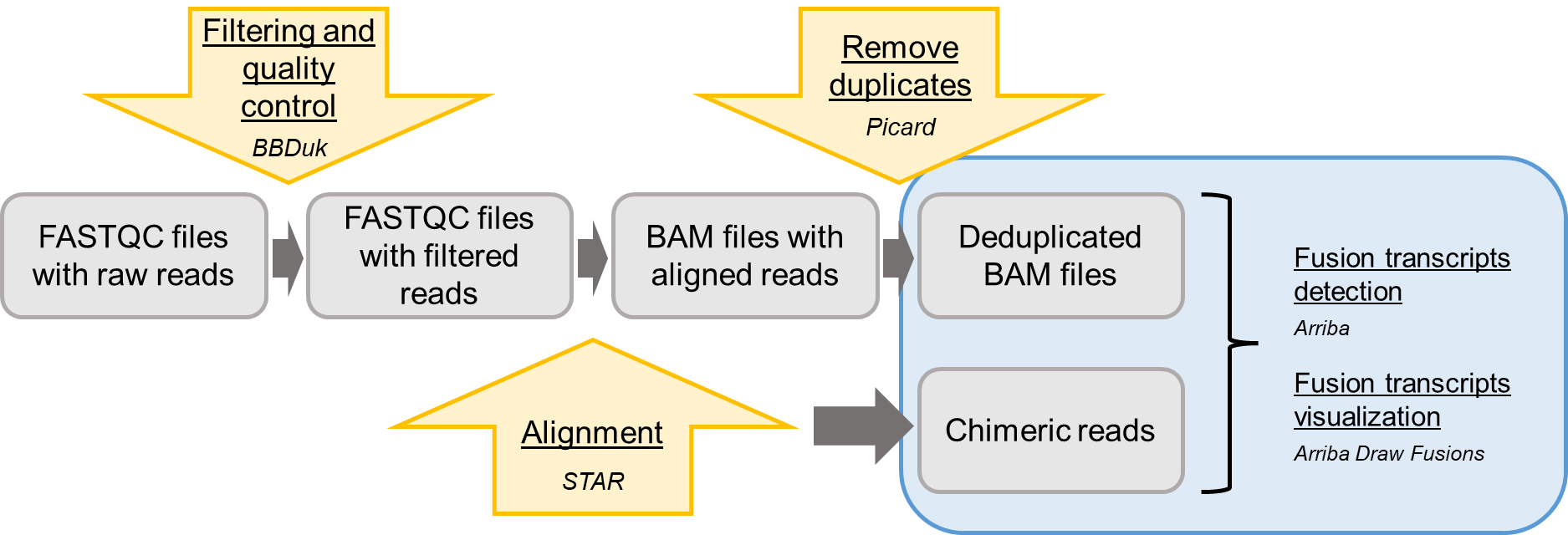


**Figure S2.** **The** **flow chart of NGS data analysis to detect fusion transcripts.**

# 2. Supplementary Tables

**Table S1.** Clinical-pathological characteristics of the study cohort. *TMERG* status is given according to the RT-qPCR results conducted before this study.

| Parameter | *TMERG*-positive (*N*=39) | *TMERG*-negative (*N*=15) |
| --- | --- | --- |
| Age at diagnosis, years |  |  |
| Mean (range) | 59 (46-82) | 61 (41-73) |
| Prostate mass, grams |  |  |
| Mean (range) | 45 (25-123) | 50 (29-90) |
| Pathological stage |  |  |
| pT2, *N* (%) | 23 (59) | 10 (67) |
| pT3, *N* (%) | 15 (38) | 5 (33) |
| pT4, *N* (%) | 1 (3) | - |
| Gleason score |  |  |
| G6, *N* (%) | 5 (13) | 2 (13) |
| G≥7, *N* (%) | 34 (87) | 13 (87) |
| Serum PSA, ng/mL |  |  |
| Mean (range) | 10.69 (2.90-33.45) | 8.71 (3.48-29.63) |
| Biochemical recurrence |  |  |
| Yes, *N* (%) | 15 (38.5) | 8 (53) |
| No, *N* (%) | 22 (56.4) | 7 (47) |
| Unknown, *N* (%) | 2 (5.1) | - |

**Table S2.** Reverse primers used in end-point PCR to validate novel breakpoints.

| Primer | Oligonucleotide sequence |
| --- | --- |
| ERG_Ex6 | 5’-GAGAAGGATGTCGGCGTTG-3’ |
| TTC18_Ex27 | 5’-CTGCTCAGCTTCTAATTGCC-3’ |
| AMACR_Ex2 | 5’-AGCTGGAGTTTCTCCATGAC-3’ |
| ERG-Linc00114 | 5’-AGACAGGTCACCATGGGTC-3’ |
| FGFR2_Ex2 | 5’-CGCTGCTGCTGCAGTC-3’ |
| OPTN_Ex5 | 5’-GCTTGATTATTTAGCTTCATGGC-3’ |
| TBXAS1_Ex14 | 5’-TTCCTCGAGGCTGCAGAAC-3’ |
| RERE_Ex14 | 5’-GTAATAGAAGGTGATCAGCTCCC-3’ |

**Table S4.** Fusion breakpoint sequences detected by FTAS-seq (*TMPRSS2* sequences underlined).

| Fusion | Breakpoint sequence |
| --- | --- |
| T1-E2 | GGGGCGGGGAGCGCCGCCTGGAGCGCGGCAGGTTATTCCAGGATCTTTGGAGACCCGAGGA |
| T1-E3 | GGGGCGGGGAGCGCCGCCTGGAGCGCGGCAGCCGTCAGGTTCTGAACAGCTGGTAGATGGG |
| T1-E4 | GGGGCGGGGAGCGCCGCCTGGAGCGCGGCAGGAAGCCTTATCAGTTGTGAGTGAGGACCAG |
| T1-E5 | GGGGCGGGGAGCGCCGCCTGGAGCGCGGCAGGAACTCTCCTGATGAATGCAGTGTGGCCAA |
| T1-E10 | GGGGCGGGGAGCGCCGCCTGGAGCGCGGCAGATCCTTATCAGATTCTTGGACCAACAAGTA |
| T2-E2 | TGTTGATAACAGCAAGATGGCTTTGAACTCAGTTATTCCAGGATCTTTGGAGACCCGAGGA |
| T2-E4 | TGTTGATAACAGCAAGATGGCTTTGAACTCAGAAGCCTTATCAGTTGTGAGTGAGGACCAG |
| T2-E5 | TGTTGATAACAGCAAGATGGCTTTGAACTCAGAACTCTCCTGATGAATGCAGTGTGGCCAA |
| T3-E4 | AAATCCCCATCCGGGACAGTGTGCACCTCAAGAAGCCTTATCAGTTGTGAGTGAGGACCAG |
| T3-E5 | AAATCCCCATCCGGGACAGTGTGCACCTCAAGAACTCTCCTGATGAATGCAGTGTGGCCAA |
| T4-E4 | GCGCTGGCCGCTGGCCTACTCTGGAAGTTCAGAAGCCTTATCAGTTGTGAGTGAGGACCAG |
| T5-E4 | TGCCCCGGCGGGGAGGACGAGAATCGGTGTGGAAGCCTTATCAGTTGTGAGTGAGGACCAG |
| T5-E5 | TGCCCCGGCGGGGAGGACGAGAATCGGTGTGGAACTCTCCTGATGAATGCAGTGTGGCCAA |
| T_Ia_-E2 | GAAAGCGGGTGTGAGGAGCGCGGCGCGGCAGGTTATTCCAGGATCTTTGGAGACCCGAGGA |
| T_Ia_-E3 | GAAAGCGGGTGTGAGGAGCGCGGCGCGGCAGCCGTCAGGTTCTGAACAGCTGGTAGATGGG |
| T_Ia_-E_IIIa_ | GAAAGCGGGTGTGAGGAGCGCGGCGCGGCAGTTAAACTCCATTGATGATGCACAGTTGACA |
| TIa-E4 | GAAAGCGGGTGTGAGGAGCGCGGCGCGGCAGGAAGCCTTATCAGTTGTGAGTGAGGACCAG |
| TIa-E5 | GAAAGCGGGTGTGAGGAGCGCGGCGCGGCAGGAACTCTCCTGATGAATGCAGTGTGGCCAA |
| T_Ib_-E_IIIc_ | TTGTCTGGGTGTGGGCATCTTATCGCCCTCTGCTTGATCTGGAATAAAGAAACAAAGGAAA |
| T1-E_IIIa_ | GGGGCGGGGAGCGCCGCCTGGAGCGCGGCAGTTAAACTCCATTGATGATGCACAGTTGACA |
| T2-E_IIIa_ | TGTTGATAACAGCAAGATGGCTTTGAACTCATTAAACTCCATTGATGATGCACAGTTGACA |
| T3-E_IIIa_ | AAATCCCCATCCGGGACAGTGTGCACCTCAATTAAACTCCATTGATGATGCACAGTTGACA |
| [T3]-E_IIIb_ | AGGGTCCTGACGCAGGCTTCCAACCCCGTCGGAATGTTTTTATATTTGGTAGAAAAATAAG |
| T3-E_IIId_ | AAATCCCCATCCGGGACAGTGTGCACCTCAAGTAATACTCAAATCAACAAATGCCCGCATT |
| T5-E_IIIa_ | TGCCCCGGCGGGGAGGACGAGAATCGGTGTGTTAAACTCCATTGATGATGCACAGTTGACA |
| T1-Linc00114 (v1) | GGGGCGGGGAGCGCCGCCTGGAGCGCGGCAGAGAGGCTCTGTGACCCATGGTGACCTGTCT |
| T2-Linc00114 (v2) | TGTTGATAACAGCAAGATGGCTTTGAACTCAAGTCTTAGTGAAGAGTGAATATTTCAAAAT |
| T1-P2 | GGGGCGGGGAGCGCCGCCTGGAGCGCGGCAGCTGTTCCATTTCCTCCAAGTCACCGGCTTA |
| T2-P2 | TGTTGATAACAGCAAGATGGCTTTGAACTCACTGTTCCATTTCCTCCAAGTCACCGGCTTA |
| T3-P2 | AAATCCCCATCCGGGACAGTGTGCACCTCAACTGTTCCATTTCCTCCAAGTCACCGGCTTA |
| T1-A2 | GGGGCGGGGAGCGCCGCCTGGAGCGCGGCAGGTGTCATGGAGAAACTCCAGCTGGGCCCAG |
| T5-A2 | TGCCCCGGCGGGGAGGACGAGAATCGGTGTGGTGTCATGGAGAAACTCCAGCTGGGCCCAG |
| T5-CASZ3 | TGCCCCGGCGGGGAGGACGAGAATCGGTGTGGATGAAGTGACACCCCCAGCTACATCCGAG |
| T5-S_VI_ | TGCCCCGGCGGGGAGGACGAGAATCGGTGTGGAAAATGGCATTTGTCGCTCTTGGCCGTCC |
| T1-TTC25 | GGGGCGGGGAGCGCCGCCTGGAGCGCGGCAGTATGAAAAGGCAAAGAAAACCTATATGCAA |
| T1-TTC26 | GGGGCGGGGAGCGCCGCCTGGAGCGCGGCAGCTGGAGGAGCTCACAGAGGCTGAGGATGCT |
| T1-TTC27 | GGGGCGGGGAGCGCCGCCTGGAGCGCGGCAGGTTGGACGGCAATTAGAAGCTGAGCAGGCC |
| T2-TTC21 | TGTTGATAACAGCAAGATGGCTTTGAACTCAGACCAGGAGCTGCATTATCTATTCTAGACA |
| T2-TTC23 | TGTTGATAACAGCAAGATGGCTTTGAACTCATATGTGCACAGAGTGCTTGCACATGAGCTG |
| T2-TTC24 | TGTTGATAACAGCAAGATGGCTTTGAACTCAAACCCCAATGTCTGGGGCCTGAAGGGCCAT |
| T1-F2 | GGGGCGGGGAGCGCCGCCTGGAGCGCGGCAGTGACTGCAGCAGCAGCGGCAGCGCCTCGGT |
| T3-O5 | AAATCCCCATCCGGGACAGTGTGCACCTCAAAAGCCATGAAGCTAAATAATCAAGCCATGA |
| T5-C2 | TGCCCCGGCGGGGAGGACGAGAATCGGTGTGACTTCCAAGTGCTGGCCTTCATCTCCCCGC |
| T1-TBX14 | GGGGCGGGGAGCGCCGCCTGGAGCGCGGCAGATGGCCCCTGAGTTCTGCAGCCTCGAGGAA |
| T1-R14 | GGGGCGGGGAGCGCCGCCTGGAGCGCGGCAGGGGGAGCTGATCACCTTCTATTACTATTGG |
| T3-R14 | AAATCCCCATCCGGGACAGTGTGCACCTCAAGGGGAGCTGATCACCTTCTATTACTATTGG |

**Table S5.** The comparison of the main technical characteristics of the RNA fusion detection methods.

|  | FTAS-seq | ArcherDx FusionPlex | QIAseq  RNAscan | Ion Torrent Oncomine | Illumina TruSight Oncology 500 |
| --- | --- | --- | --- | --- | --- |
| Recommended total RNA input amount | 500 ng | 200 ng | 50 ng | 20 ng | 40 ng |
| Compatible with degraded RNA | Yes | Yes | Yes | Yes | Yes |
| Enrichment approach | Semi-targeted amplification | Semi-targeted amplification | Semi-targeted amplification | Targeted amplification | Hybridization capture |
| Detection of novel fusions | Yes | Yes | Yes | No | Yes |
| Sample preparation time | 1 day | 2 days | 2 days | 2 days | 2 days |
