# Supplementary material for: Fusion sequencing via terminator‐assisted synthesis (FTAS‐seq) identifies TMPRSS2 fusion partners in prostate cancer
Source: Mol Oncol. 2023 Apr 13;17(6):993–1006. doi: 10.1002/1878-0261.13428 (PMC10257418; doi:10.1002/1878-0261.13428)
Supplement: Supplementary file 2 — Table S3. The variety of fusion transcripts detected in individual patient samples by FTAS‐seq, RT‐qPCR and Sanger sequencing. [file MOL2-17-993-s001.pdf]

Supplementary Data

Table S3. The variety of fusion transcripts detected in individual patient samples by FTAS-seq, RT-qPCR and Sanger sequencing. Asterisk (*) marks *TMERG* variants detected by RT-qPCR. H, M, L corresponds to fusion transcripts detected by FTAS-seq with high, medium, or low confidence scores, respectively. Yellow cells mark variants detected by FTAS-seq. Green cells mark variants detected by Sanger sequencing.

			Individual prostate tissue RNA samples																																																					
			1	2	3	4	5	6	7	8	9	10	11	12	13	14	15	16	17	18	19	20	21	22	23	24	25	26	27	28	29	30	31	32	33	34	35	36	37	38	39	40	41	42	43	44	45	46	47	48	49	50	51	52	53	54
Detected by NGS	*TMPRSS2-ERG* wild type	N/A																													wt		wt		wt	wt			wt	wt			wt	wt	wt	wt				wt			wt	wt		
	TMPRSS2-ERG	T1-E2		H*					M															*				H*	M	H							H																			
	TMPRSS2-ERG	T1-E3		H																								H	M	M							H																			
	TMPRSS2-ERG	T1-E4	H*	H	M*	M*	H*	M*	M*	H*	H*	M*	M*	H*	M*	M*	M*	M*	*	M*	H*	H	H*	H	H*	H*	H*	M	M*	H*		H		*			H*	M*			H*	H*					L*	H*	*		H*	H*			H*	*
	TMPRSS2-ERG	T1-E5	H		M		H	M	M	H	M	M	M		M	M	M	M		M	M	M	M	H	H	H	M	M	M	M		M					H	M			H	H					L	M			M	H			M	
	TMPRSS2-ERG	T1-E10																																																		M				
	TMPRSS2-ERG	T2-E2							H																			H																												
	TMPRSS2-ERG	T2-E4				H*		H*	H*	M				H						H*	H	H					H		*	H*				*				H*									*								H*	*
	TMPRSS2-ERG	T2-E5							H																																															
	TMPRSS2-ERG	T3-E4												H								H																H									H									
	TMPRSS2-ERG	T3-E5																				M																H									H									
	TMPRSS2-ERG	T4-E4																																															H							
	TMPRSS2-ERG	T5-E4																																															H							
	TMPRSS2-ERG	T5-E5																																															H							
	TMPRSS2-ERG	TIa-E2		M																								H	H								H																			
	TMPRSS2-ERG	TIa-E3																										M																												
	TMPRSS2-ERG	TIa-EIIIa																						H	H	H																H						H				M				
	TMPRSS2-ERG	TIa-E4	H		H		H			H	H							H				H	H	H	H	H	H		H	H		H					H	M			H	H						H			H	H			H	
	TMPRSS2-ERG	TIa-E5																					M																		M											M				
	TMPRSS2-ERG	TIb-EIIIc														M																																								
	TMPRSS2-ERG	T1-EIIIa	H		M		H	M	L			M	M	H	M	M	M			L	H	H	H	H	H	H			M	H							H				H	H					L	H			H	H				
	TMPRSS2-ERG	T2-EIIIa						H	H					H																H																										
	TMPRSS2-ERG	T3-EIIIa																																													H									
	TMPRSS2-ERG	[T3]-EIIIb				M																																																		
	TMPRSS2-ERG	T3-EIIId																				M																																		
	TMPRSS2-ERG	T5-EIIIa																																															H							
	TMPRSS2-ERG,Linc00114	T1-ERG,Linc00114 (v1)																											M																											
	TMPRSS2-ERG,Linc00114	T2-ERG,Linc00114 (v2)																										M																												
	TMPRSS2-PPP3CA	T1-P2																	M																																					
	TMPRSS2-PPP3CA	T2-P2																	H																																					
	TMPRSS2-PPP3CA	T3-P2																	H																																					
	TMPRSS2-AMACR	T1-A2																				M		M	M	M				M											L	M		M												
	TMPRSS2-AMACR	T5-A2																				H																																		
	TMPRSS2-CASZ1	T5-CASZ3																						H																																
	TMPRSS2-SIM2	T5-SVI																				H																																		
	TMPRSS2-TTC18	T1-TTC25																				L																																		
	TMPRSS2-TTC18	T1-TTC26																				M																																		
	TMPRSS2-TTC18	T1-TTC27																				M																																		
	TMPRSS2-TTC18	T2-TTC21																				M																																		
	TMPRSS2-TTC18	T2-TTC23																				L																																		
	TMPRSS2-TTC18	T2-TTC24																				L																																		
	TMPRSS2-FGFR2	T1-F2																																			M																			
	TMPRSS2-OPTN	T3-O5																																						H																
	TMPRSS2-C1orf61	T5-C2																																						H																
	TMPRSS2-TBXAS1	T1-T14																																											M											
	TMPRSS2-RERE	T1-R14																																																				M		
	TMPRSS2-RERE	T3-R14																																																				H		
Detected by Sanger	*TMPRSS2-ERG*	T1-E6																																																						
	TMPRSS2-ERG	T2-E6																																																						
	TMPRSS2-ERG	T3-E6																																																						
	TMPRSS2-ERG	T4-E5																																																						
	TMPRSS2-ERG,Linc00114	T2-ERG,Linc00114 (v1)																																																						
	TMPRSS2-AMACR	T2-A2																																																						
	TMPRSS2-TTC18	T2-T26																																																						
	TMPRSS2-TTC18	T2-T27																																																						
	TMPRSS2-FGFR2	T1-FI																																																						
	TMPRSS2-OPTN	T1-O5																																																						
	TMPRSS2-OPTN	T2-O5																																																						
	TMPRSS2-TBXAS1	T2-TBX14																																																						
	TMPRSS2-RERE	T2-R14																																																						
